# Selecting short-statured children needing growth hormone testing: Derivation and validation of a clinical decision rule

**DOI:** 10.1186/1471-2431-8-29

**Published:** 2008-07-16

**Authors:** Laëtitia Duché, Christine Trivin, Wassim Chemaitilly, Jean Claude Souberbielle, Gérard Bréart, Raja Brauner, Martin Chalumeau

**Affiliations:** 1 Clinical Epidemiological Unit-Department of Pediatrics, Saint-Vincent-de-Paul Hospital, AP-HP, Université Paris Descartes, 75014 Paris, France; 2 INSERM U149, 75014 Paris, France; 3 Université Paris Descartes and Assistance Publique Hôpitaux de Paris, Hôpital Bicêtre, Unité d'endocrinologie pédiatrique, 94270 Le Kremlin Bicêtre, France; 4 Assistance Publique Hôpitaux de Paris, Necker-Enfants Malades Hospital, Service d'explorations fonctionnelles, 75743 Paris, France

## Abstract

**Background:**

Numerous short-statured children are evaluated for growth hormone (GH) deficiency (GHD). In most patients, GH provocative tests are normal and are thus in retrospect unnecessary.

**Methods:**

A retrospective cohort study was conducted to identify predictors of growth hormone (GH) deficiency (GHD) in children seen for short stature, and to construct a very sensitive and fairly specific predictive tool to avoid unnecessary GH provocative tests. GHD was defined by the presence of 2 GH concentration peaks < 10 ng/ml. Certain GHD was defined as GHD and viewing pituitary stalk interruption syndrome on magnetic resonance imaging. Independent predictors were identified with uni- and multi-variate analyses and then combined in a decision rule that was validated in another population.

**Results:**

The initial study included 167 patients, 36 (22%) of whom had GHD, including 5 (3%) with certain GHD. Independent predictors of GHD were: growth rate < -1 DS (adjusted odds ratio: 3.2; 95% confidence interval [1.3–7.9]), IGF-I concentration < -2 DS (2.8 [1.1–7.3]) and BMI z-score ≥ 0 (2.8 [1.2–6.5]). A clinical decision rule suggesting that patients be tested only if they had a growth rate < -1 DS and a IGF-I concentration < -2 DS achieved 100% sensitivity [48–100] for certain GHD and 63% [47–79] for GHD, and a specificity of 68% [60–76]. Applying this rule to the validation population (n = 40, including 13 patients with certain GHD), the sensitivity for certain GHD was 92% [76–100] and the specificity 70% [53–88].

**Conclusion:**

We have derived and performed an internal validation of a highly sensitive decision rule that could safely help to avoid more than 2/3 of the unnecessary GH tests. External validation of this rule is needed before any application.

## Background

Shortness or decreasing growth is a frequent reason for pediatric consultations. After ruling out other causes of short stature (intestinal malabsorption, chronic liver or kidney disease, hypothyroidism, etc), the possibility of growth hormone (GH) deficiency (GHD) is often considered. This deficiency is associated with excess mortality and substantial morbidity [1,2], and it can be treated. Many children are therefore referred by their physicians to specialist departments to test for GHD. Testing is based on the measurement of stimulated GH secretion [3,4]: the diagnosis is generally based on 2 GH peaks < 10 ng/mL (or 20 mUI/mL)[4]. GHD cannot be considered certain unless there are also one or more of the following confirmatory markers: familial GHD, other deficiency of the hypothalamic-pituitary axis, micropenis, neonatal hypoglycemia, abnormalities of the median line and pituitary stalk interruption syndrome (PSIS) on magnetic resonance imaging (MRI) [5].

GH stimulation tests are invasive, expensive, and in view of the risk of severe hypoglycemia [6], potentially dangerous [7]. Moreover they are normal in most cases and thus retrospectively unnecessary. It would therefore be useful to be able to identify predictive factors of GHD to avoid these unnecessary tests. A selection strategy for GH stimulation tests, however, must offer sensitivity close to 100% for certain GHD, in view of the need to begin treatment rapidly [8]; it must also be sufficiently specific.

Clinical (height, growth rate, difference between height and midparental target height) [3] and laboratory (insulin-like growth factor-I IGF-I]) [3,9] criteria have been proposed to predict GHD. Used separately, these different criteria do not fulfill the objectives described above. It may therefore be useful to combine them. Earlier clinical decision rules have proposed combining clinical and laboratory variables [10,11] to avoid GH stimulation tests. One rule combined growth rate and IGF-I [10], and the other chronological age, bone age, body mass index (BMI) and IGF-I [11]. Nonetheless the results of these studies are limited by selection bias in patient recruitment [10], the absence of multivariate analyses despite the very probable correlations between variables [10], the complexity of the calculations necessary to apply the rule [11], and insufficient predictive performance [10]. This is probably why none of these tools has undergone internal or external validation.

The objective of this study was therefore to identify the predictive factors for GH deficiency in children consulting for short stature and/or decreased growth rate and to construct and validate internally a very sensitive and fairly specific predictive tool that is simple to use to avoid unnecessary tests.

## Methods

### Patients

This was a retrospective hospital-based cohort study. All patients were seen by a senior pediatric endocrinologist (RB) from January 1998 through June 2001 at Necker-Enfants Malades Hospital in Paris, France. The Ethical Review Committee (Comité de Protection des Personnes Ile de France III) stated that "this research was found to conform to generally accepted scientific principles and research ethical standards and to be in conformity with the laws and regulations of the country in which the research experiment was performed" (see Additional file 1). Written informed consent of the patients or their parents was not judged necessary for that kind of retrospective study.

The patients included were 1 to 16 years-old and had at least one of the principal auxological criteria for which the GH Research Society consensus conference guidelines require GH stimulation testing [3] (height ≤ -3 standard deviations (SD), growth rate ≤ -2 SD for chronological age, or height ≤ -2 SD, growth rate ≤ -1 SD, and a difference between current height and midparental target height > 1.5 SD). They had also had 2 tests assessing GH secretion: one of spontaneous secretion during sleep and one after pharmacological stimulation.

We excluded from this study the patients with conditions other than GHD that were responsible for their short stature (hypothyroidism, celiac disease, gastrointestinal inflammatory disease, cystic fibrosis, kidney failure, or Turner syndrome) as well as those for which GHD was due to a condition already known at the consultation (lesion, surgery and/or irradiation of the hypothalamic-pituitary region) and those with signs and findings highly suggestive of GHD: familial GHD, history or clinical picture suggesting pituitary deficiency (polyuric-polydipsic syndrome, severe hypoglycemia in the first months of life, micropenis, abnormalities of the median line). Indeed, for these high-risk patients, there is no need for a selective strategy. Patients who had had testosterone or estradiol priming and those with delayed puberty (defined by a Tanner stage of 1 for a girl older than 13 years or a boy older than 14) were also excluded.

### Predicted variable

The variable to be predicted was GHD. Plasma GH (hGH immunoradiometric assay, Immunotech, Marseille, France) was measured for each patient from blood samples taken while sleeping (samples every 30 minutes from 22 h to 6 h) followed in the morning by a provocative test administering arginine and insulin sequentially (arginine 0.5 g/kg intravenous perfusion for 30 min; insulin at 60 min 0.1 U/kg intravenously, n = 64), ornithine (HCl 14.5 g/m^2 ^intravenous perfusion for 30 min, n = 73) or glucagon (0.1 mg/kg intramuscular injection, 1 mg maximum, n = 30). During the study period, the treatment protocol called for MRI if the 2 GH peaks were less than 10 ng/mL, to look for PSIS (thin or interrupted stalk, ectopic posterior or hypoplasic anterior pituitary gland [12]).

Children were then classified in 2 groups as a function of the GH assay and MRI results: no GHD (1 GH peak ≥ 10 ng/mL) or GHD (2 GH peaks < 10 ng/mL). Within the GHD group, children with pituitary stalk interruption syndrome on MRI were considered to have certain GHD, and the other patients were considered to have uncertain GHD.

### Potential predictors

The following potential clinical predictors were studied: chronological age expressed in years, height measured with a Harpenden stadiometer and expressed in SD, growth rate expressed in SD [13], BMI measured as weight in kilograms divided by the square of height in meters and expressed as a z-score compared with chronological age [14], difference in SD between height and the midparental target height, calculated from both parents' height [15], and pubertal stage (breast or testes) [16,17]. Two potential non clinical predictors were also studied: plasma IGF-I (IGF-I-RIACT, Cis Bio, Gif sur Yvette, France) expressed in SD according to chronological age [18] and bone age delay (difference in years between chronological age and bone age) [19].

### Analysis

STATA/SE 8 (Statacorp, College Station, TX, USA) software was used for the statistical analysis. We began by using the Mann-Whitney test to compare the distribution of the possibly predictive continuous variables as a function of GHD. Next, the continuous variables were dichotomized, either according to the standard cutoff point in the literature or according to their distribution in patients without GHD (median or one of the quartiles rounded to the nearest half point). For "pubertal stage", the last 4 Tanner stages were combined into one to obtain a reproducible variable (prepubertal versus pubertal children). We conducted a bivariate analysis to study the relation between GHD and the dichotomized variables and calculate odds ratios. Comparisons were tested with the Chi-2 test or Fisher's exact test. Next, we used logistic regression to conduct a multivariate analysis.

### Decision rule derivation

First, the discriminant power of the independent variables associated with GHD was studied by the calculation of their sensitivity, specificity, positive predictive value and negative predictive value for GHD and for certain GHD. To meet our objective of high sensitivity (close to 100%) for certain GHD with the best possible specificity (around 2/3), we varied the cutoff points of the independent predictors. Since no independent predictor used alone met these objectives, we then combined them by recursive partition to construct a decision rule, along the lines of previous rules for pediatric endocrinology [20,21]. To make the tool simple for clinicians to use, we chose only whole values close to the standard thresholds to dichotomize the variables.

### Decision rule validation

The predictive tool was validated among 2 populations of consecutive patients meeting the inclusion criteria described above: a population of patients with certain GHD seen from 1990–1998 and 2001–2005 and a population of patients seen in 2002 with 1 GH peak ≥ 10 ng/mL and no cause for short stature found, and thus considered not GH-deficient. The data for the validation populations remained blinded during construction of the rule, and the rule was not modified after application to these populations.

## Results

The analysis included 167 children. Their mean age was 8.2 years (range 1.1–15.5; interquartile range 5.1–11.3) and 49% were boys; 36 (22%) children diagnosed with GHD, including 5 (3%) with certain GHD.

### Predictive variables

Patients with GHD (Table 1) had a lower growth rate, higher BMI, and lower IGF-I level than the patients without GHD (p < 0.05). No statistically significant (p > 0.10) difference was shown in the distribution of age, height, difference from midparental target height, weight or bone age delay between the two groups. After dichotomization (Table 2), there was a statistically significant association between GHD and growth rate < -1 SD (p = 0.005) as well as BMI z-score ≥ 0 (p = 0.006). A trend that did not reach statistical significance was seen between GHD and both a prepubertal Tanner stage (p = 0.09) and IGF-I < -2 SD (p = 0.09). No statistically significant association was observed with age < 5 years, height < -2.5 SD, height difference with midparental target height ≥ -3 SD, weight ≥ -2 SD (p > 0.20) or delayed bone age ≥ 1.5 years (p > 0.20).

**Table 1:** Distribution of potential predictors among children with or without GHD.

**Variables**				**GHD**						p**
					
	**Absence of GHD**			**Uncertain**			**Certain**			
				
	**n**	**mean**	SD*	**n**	**mean**	SD*	**n**	**mean**	SD*	
**History and physical examination**										
Age (years)	131	8.3	3.8	31	8.1	3.6	5	5.2	2.4	0.4
Height (SD)	131	-2.2	0.6	31	-2.4	0.7	5	-2.3	0.5	0.2
Weight (SD)	131	-1.7	0.8	31	-1.3	1.2	5	-1.3	1.4	0.07
Growth rate (SD)	127	-0.9	1.6	30	-1.5	1.0	5	-2.3	0.9	0.01
Difference between height and MTH***	127	-2.1	0.9	29	-2.1	1.1	4	-3.1	0.3	0.7
BMI (z-score)	131	-0.8	1.1	31	0.0	1.4	5	0.0	2.2	0.001
										
**Studies**										
IGF-I (SD)	131	-2.2	1.3	31	-2.7	1.1	5	-3.9	1.1	0.03
Bone age delay (years)	125	2.1	1.1	29	2.0	0.9	5	2.2	0.9	0.9

* Standard deviation

** Non parametric Mann-Whitney U-test (between patients with and without GHD)

*** Midparental target height

**Table 2:** Relations between potential predictors and GHD, before and after adjustment.

**Variables**	Absence of GHD (n = 131)		GHD (n = 36)		OR	CI	p	AOR*	CI*	pAOR*
								
	n	%	n	%						
**History and physical examination**										
Boys	62	47	20	56	1.4	0.7–2.9	0.4			
Girls	69	53	16	44						
										
Age < 5 years	29	22	10	28	1.4	0.6–3.1	0.5			
Age ≥ 5 years	102	78	26	72						
										
Height < -2.5 SD	48	37	16	44	1.4	0.7–2.9	0.4			
Height ≥ -2.5 SD	83	63	20	56						
										
Weight ≥ -2 SD	21	16	8	22	1.5	0.6–3.8	0.4			
Weight < -2 SD	110	84	28	78						
										
Growth rate < -1 SD	64	50	27	77	3.3	1.4–8.1	0.005	3.2	1.3–7.9	0.01
Growth rate ≥ -1 DS	63	50	8	23						
										
Difference between height and MTH ≤ -3 SD	15	12	5	15	1.3	0.4–4.0	0.6			
Difference between height and MTH > -3 SD	112	88	28	85						
										
BMI ≥ 0 z-score	31	24	17	47	2.9	1.3–6.4	0.006	2.8	1.2–6.5	0.01
BMI < 0 z-score	100	76	19	53						
										
Pre-pubertal children (Tanner = 1)	104	79	33	92	2.9	0.8–10	0.09	**		
Pubertal children (Tanner > 1)	27	21	3	8						
										
**Studies**										
IGF-I < -2 SD	82	63	28	78	2.1	0.9–5.0	0.09	2.8	1.1–7.3	0.04
IGF-I ≥ -2 SD	49	37	8	22						
										
Bone age delay ≥ 1.5 years	88	70	27	79	1.6	0.6–4.1	0.3			
Bone age delay < 1.5 years	37	30	7	21						

* OR adjusted for variables with a p < 0.1 in bivariate analysis (N = 162)

** Variable excluded from the model

*** Midparental target height

After adjustments, GHD was not significantly (p > 0.05) associated with a prepubertal stage (Table 2), but was significantly and independently associated with growth rate < -1 SD, BMI z-score ≥ 0 and IGF-I < -2 SD.

### Decision rule

**Figure 1:**
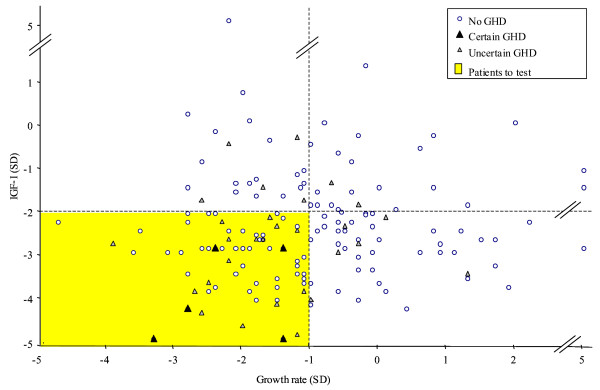
Patients distribution according to their growth rate (SD) and IGF-I level (SD).

None of the criteria used alone allowed us to reach the objectives we had set: sensitivity of 100% for certain GHD and specificity ≥ 2/3 (Table 3). The best combination of predictive independent variables was growth rate and IGF-I (Figure 1). A clinical decision rule suggesting that GH stimulation testing was necessary only if these 2 indicators (growth rate < -1 SD and IGF-I < -2 SD) were both present yielded a specificity of 68% (95% CI [60–76]) with a sensitivity of 100% (95% CI [48–100]) for the certain GHD diagnosis. Adding BMI to this combination in a decision tree or composite score did not improve its predictiveness. Of the patients with uncertain GHD, 43% were not identified by the rule. These patients had a mean age of 8.5 years, a mean height of -2.3 SD, a mean growth rate of -0.8 SD and a mean IGF-I of -2.0 SD. None had panhypopituitarism and 84% had not had GH treatment.

**Table 3:** Predictive values of GHD predictors.

							Clinical decision rule***		
							
	**Growth rate < -1 SD**		**BMI ≥ 0 z-score**		**IGF-I < -2 SD**		**Construction†**		**Validation‡**
					
**GHD**	**All**	**Certain**	**All**	**Certain**	**All**	**Certain**	**All**	**Certain**	
Sensitivity	77 [60–90]	100 [48–100]	47 [31–63]	60 [15–95]	78 [64–91]	100 [48–100]	63 [47–79]	100 [48–100]	92 [76–100]
Specificity	50 [41–58]	Idem	76 [69–83]	Idem	37 [29–46]	Idem	68 [60–76]	Idem	70 [53–88]
PPV*	30 [20–39]	7 [2–16]	35 [22–48]	9 [2–24]	25 [17–34]	6 [2–13]	35 [23–47]	11 [2–20]	Not applicable
NPV**	89 [79–95]	100 [94–100]	84 [77–91]	98 [95–100]	86 [77–95]	100 [93–100]	87 [80–94]	100 [92–100]	Not applicable

Values are expressed in % [95% CI]

* Positive predictive value

** Negative predictive value

*** To test patients with growth rate < -I SD and IGF-I < -2 SD

† Construction population

‡ Validation population

For the periods 1990–1998 and 2001–2005, 13 patients who met the inclusion criteria had certain GHD. The sensitivity of the combination of growth rate < -1 SD and IGF-I level < -2 SD was 92% (95% CI [76–100]). The one patient with certain GHD who was not identified by the rule was a 13-year-old boy with a height -2.9 SD and a growth rate of -0.9 SD; he had no other pituitary deficiencies and was treated with GH. In 2002, 27 patients had at least one GH peak ≥ 10 ng/mL and met the inclusion criteria. The specificity of the predictive tool applied to this population was 70% (95% CI [53–88]).

## Discussion

Three independent predictive factors were identified among the patients we studied: growth rate < -1 SD, IGF-I < -2 SD and BMI z-score ≥ 0. Growth rate is a classic predictor of GHD. Different cutoff points have been proposed in the literature [22-25] including the one we used here (< -1 SD). The predictive power of the IGF-I level has been studied repeatedly [5,9,25-29]. The results in terms of sensitivity and specificity vary widely, but this assay is very useful for the diagnosis of GHD [9]. The cutoff point we used (-2 SD) is that usually found in the literature.

In our study, a BMI z-score ≥ 0 was also an independent predictive factor of GHD. This criterion is most often considered a confounding factor instead [4]. That is, on the one hand, children with simple obesity have an abnormally low response to GH stimulation tests and on the other hand, some children with GHD have truncal obesity. Accordingly a predictive tool that uses this criterion might therefore be dangerous. Moreover, it does not improve the rule's predictive power.

The clinical decision rule we propose here is that GH stimulation tests should be performed only on children with a growth rate of < -1 SD and an IGF-I level < -2 SD. These variables were also included in the rule proposed by Cianfarani et al but with a different combination [10]. Our decision rule has good clinical applicability because it uses predictive variables at the rounded cutoff points already used by clinicians. Moreover, it is probably robust because it uses independent predictors identified by multivariate analysis. This rule should make it possible to avoid two thirds of the GH stimulation tests that are retrospectively unnecessary because normal, while missing in our series only one of 18 cases of certain GHD. This patient had an IGF-I level < -2 SD but a growth rate of -0.9 SD. It is probable that he would have reached -1 SD during his next follow-up, thus being "caught" by the rule. Moreover since he did not have panhypopituitarism, there was no immediate metabolic danger [1]. Our rule is a relatively insensitive predictive tool for the diagnosis of uncertain GHD: 43% of these patients were not identified. Compensating for this poor prediction is the fact that these patients did not have panhypopituitarism, that 84% of them did not receive treatment, and that the abnormal character of uncertain GHD is currently the subject of much debate [30,31]. Indeed, GH secretion in most children with a subnormal GH response to GH stimulation tests but normal MRI becomes normal when they are retested at the completion of growth or even after few months. Thus, it is likely that many of the patients in the uncertain GHD group did not have GHD. Some of them have been retested (16%) and had normal GH secretion; they may be considered to have had transient GHD. Others reached a normal final height without treatment (7%). The other patients are still being closely monitored for their growth velocity.

Some of the patients (n = 5, 16%) with uncertain GHD, and a IGF-I concentration ≥ -2 SD but a growth rate < -1 SD, would not have been identified as requiring GH secretion evaluation according to the decision rule (Figure 1). This potential false-negative rate shows the need for follow-up of the growth of these patients not identified as requiring GH secretion evaluation.

There were two potential sources of bias in our study. First, patients came from a specialist pediatric endocrinology outpatient department and were very probably at higher risk of GHD than any other population. This bias is demonstrated by the very high prevalence (22%) of patients with GHD compared with other series [11]. We also excluded children with priming with testosterone or estradiol to avoid a confounding bias with IGF-I, because we were treating it as a potential predictive variable and priming increases both IGF-I and peak GH concentrations [32]. Second, there may have been classification errors for the predicted variable. That is, although stimulation tests must be used to evaluate short children for whom GHD is considered, their reproducibility is poor. Response varies substantially according to the stimulus used [33]. Moreover, test response also includes a component of intraindividual variability. In a study of 40 Israeli children, Zadik and coll. reported a moderate correlation (r = 0.52) between 2 tests conducted 6 weeks apart with the same pharmacological agent [34]. In our study, as in all studies that have used stimulation tests as the reference test, classification errors may intervene between the diagnosis of GHD (defined by 2 GH peaks < 10 ng/mL without a confirmation criterion) and no GHD. Errors for the certain cases are less plausible because the criterion of certain GHD (pituitary MRI) is more robust. Furthermore, when we used a stimulation test cut-off of 7 ng/ml instead of 10 ng/ml to define GHD, the decision rule's sensitivity was 67% (95% CI [43–91]) (vs 63% (95% CI [47–79])) for patients with GHD and did not change for patients with certain GHD (100% (95% CI [48–100])). The specificity was 64% (95% CI [56–71]) (vs 68% (95% CI [60–76])).

## Conclusion

The suboptimal nature of a systematic strategy of stimulation tests and the intrinsic limitations of these tests make the construction of a predictive tool for GHD necessary. The tool we propose is very effective for certain GHD but far less so for uncertain GHD. The current debate about the abnormal character of uncertain GHD [30,31] highlights the interest of our tool. Nonetheless, in view of the limitations of our study and especially the low number of patients with certain GHD, these results should be validated at other centers, as other decision rules in pediatric endocrinology have been [21], before any widespread clinical application.

## Abbreviations

GH – Growth hormone; GHD – Growth hormone deficiency; PSIS – Pituitary stalk interruption syndrome; MRI – Resonance magnetic imaging; IGF-I – Insulin-like growth factor-I; BMI – Body mass index; SD – Standard deviation.

## Competing interests

The authors declare that they have no competing interests.

## Authors' contributions

All the authors 1) have made substantial contributions to conception and design, or acquisition of data, or analysis and interpretation of data; 2) have been involved in drafting the manuscript or revising it critically for important intellectual content; and 3) have given final approval of the version to be published.

## Pre-publication history

The pre-publication history for this paper can be accessed here:



## Supplementary Material

Additional file 1 Ethical Review Committee statement. Ethical Review Committee statement of the Comité de Protection des Personnes Ile de France III. Click here for file
