# Supplementary material for: Selecting short-statured children needing growth hormone testing: Derivation and validation of a clinical decision rule
Source: BMC Pediatr. 2008 Jul 16;8:29. doi: 10.1186/1471-2431-8-29 (PMC2492843; doi:10.1186/1471-2431-8-29)
Supplement: Additional file 1 — Ethical Review Committee statement. Ethical Review Committee statement of the Comité de Protection des Personnes Ile de France III. [file 1471-2431-8-29-S1.pdf]

COMITE DE PROTECTION DES PERSONNES
ILE-DE-FRANCE III

Hôpital TARNIER-COCHIN
89, rue D'Assas , 75006 Paris

Secrétariat : Tél : 33 (1) 46-33-68-67
Fax : 33 (1) 46-33-70-46
E-mail : cpp.iledefrance3@orange.fr

Paris le 24 juin 2008

This is to certify that:

1/ The Ethical Review Committee «Comité de Protection des Personnes Ile de France III » has examined the research entitled ***« Selecting short-statured children needing growth hormone testing : derivation and validation of a clinical decision rule».***

2/ This research was found to conform to generally accepted scientific principles and research ethical standards.

3/ This research was found to be in conformity with the laws and regulations of the country in which the research experiment was performed.

President :

Professeur Boyan Christoforov
